# Improving maternity care using a personal health record: study protocol for a stepped-wedge, randomised, controlled trial

**DOI:** 10.1186/s13063-016-1326-0

**Published:** 2016-04-16

**Authors:** Carola J. M. Groenen, Marjan J. Faber, Jan A. M. Kremer, Frank P. H. A. Vandenbussche, Noortje T. L. van Duijnhoven

**Affiliations:** Department of Obstetrics and Gynaecology, Radboud university medical center, P.O. Box 9101, 6500 HB Nijmegen, The Netherlands; Scientific Institute for Quality of Healthcare (IQ healthcare), Radboud university medical center, P.O. Box 9101, 6500 HB Nijmegen, The Netherlands

**Keywords:** Personal Health Record, Maternity care, Protocol, Stepped-wedge, cluster-randomised, controlled trial

## Abstract

**Background:**

A personal health record (PHR) is an online application through which individuals can access, manage, and share their health information in a private, secure, and confidential environment. Personal health records empower patients, facilitate collaboration among healthcare professionals, and improve health outcomes. Given these anticipated positive effects, we want to implement a PHR, named MyPregn@ncy, in a Dutch maternity care setting and to evaluate its effects in routine care. This paper presents the study protocol.

**Methods/design:**

The effects of implementing a PHR in maternity care on patients and professionals will be identified in a stepped-wedge, cluster-randomised, controlled trial. The study will be performed in the region of Nijmegen, a Dutch area with an average of 4,500 births a year and more than 230 healthcare professionals involved in maternity care. Data analyses will describe the effects of MyPregn@ncy on health outcomes in maternity care, quality of care from the patients’ perspectives, and collaboration among healthcare professionals. Additionally, a process evaluation of the implementation of MyPregn@ncy will be performed. Data will be collected using data from the Dutch perinatal registry, questionnaires, interviews, and log data.

**Discussion:**

The study is expected to yield new information about the effects, strengths, possibilities, and challenges to the implementation and usage of a PHR in routine maternal care settings. Results may lead to new insights and improvements in the quality of maternal and perinatal care.

**Trial registration:**

Netherlands Trial Register: NTR4063

## Background

Personal health records (PHRs) have been embraced widely in the last decade. PHRs exist in various types, but their shared goal is to be an online application through which individuals can access, manage, and share their health information in a private, secure, and confidential environment. PHRs have been reported to empower patients, to achieve better cooperation among healthcare professionals, and to improve health outcomes [1–4]. The intended benefits of a PHR, which include the achievement of higher standards of care, are welcome in any healthcare setting.

The active involvement of the pregnant woman and better collaboration among the healthcare professionals are explicitly mentioned in the leading Dutch report [5] as components of a strategy to speed up a reduction in Dutch perinatal mortality [6]. Better multidisciplinary collaboration is associated with better quality and outcomes of healthcare delivery [7]. Given the documented positive effects of a PHR and the identified challenges in Dutch maternity care, a PHR in maternity care might be a possible tool to deal with these challenges. Therefore, we will introduce a PHR named MyPregn@ncy. We designed a study to implement MyPregn@ncy in routine maternal care and to evaluate the effects and adoption of MyPregn@ncy by pregnant women and healthcare professionals. We will identify the effects of MyPregn@ncy on the health outcomes in maternity care, quality of care from patients’ perspectives, and collaboration among healthcare professionals. Additionally, we will focus on the process of implementation of this innovative and complex intervention. We hypothesise that the introduction of a PHR for pregnant women improves health outcomes, positively influences the quality of care from patients’ perspectives, and enhances collaboration among healthcare professionals. We believe it is feasible to implement such an apocalyptical PHR in Dutch maternity care, which will eventually lead to the improvement of maternal and perinatal care.

The aim of this paper is to present an overview of the study. We describe the development of the tool MyPregn@ncy, the design of the study, and the specific aspects of evaluation.

## Methods/design

### Setting

In Dutch maternity care, different healthcare professionals are involved with a pregnant woman and her (unborn) child [8]. Community-based midwives, maternity care assistants, youth health physicians, and nurses provide primary care. Obstetricians, obstetricians in training, hospital-based midwives, and paediatricians provide secondary and tertiary care. Community-based midwives are qualified to provide full prenatal and perinatal care to all women with uncomplicated pregnancies and childbirths. In case of risk factors or complications, women are referred to secondary or tertiary care [9]. Tertiary care takes place in centres for perinatology with a neonatal intensive care unit and an obstetric ‘high-care’ department.

The hospital-based midwife is supervised by the obstetrician (in training) and attends approximately one-half of the births in secondary/tertiary care [10]. The maternity care assistant supports the community-based midwife during childbirth and subsequently takes care of the mother and child up to day 10 postpartum at the home of the new-born. She reports essential health information to the community-based midwife, who is responsible for medical care. Afterwards, the youth health department takes over the care for the baby, with the support of specialised doctors and nurses.

In 2014, 86 % of all pregnant women started their maternity care in a primary care setting. Of these women, 51 % started and 29 % eventually completed birth in a primary care setting [10]. After birth, the medical care is under the supervision of a community-based midwife, and 96 % of all women later receive care from a maternity care assistant at home [10]. Overall, the Dutch system involves a number of referrals during pregnancy, during birth, and after birth.

Healthcare professionals in maternity care work in a regional multidisciplinary collaborative organization situated around a hospital or city, in which they create joint protocols and make distinct appointments for referrals in (acute) care.

### Study population

The study will be performed in one regional collaborative organisation in the area of Nijmegen, a Dutch region with an average of 4,500 births a year and more than 230 healthcare professionals involved in maternity care. Community-based midwives are employed in one of 11 independent practices, whereas hospital-based midwives, obstetricians (in training), and paediatricians work in two different hospitals (one providing secondary care and one providing secondary and tertiary care). Maternity care assistants are employed by one organisation and youth health doctors and nurses are employed by one of 14 offices, all of which are coordinated by one organisation.

Each pregnant woman, independently of the gestational age and care setting, will be invited to participate in this study individually; that is, she will be offered the possibility to start her PHR (MyPregn@ncy). All healthcare professionals in the area will be informed of and requested to actively participate in the study.

### Ethical approval

The medical ethical committee of the Radboud University Medical Centre has awarded full ethical approval for this project (CMO No. 2011/381). The study is registered at the Dutch Trial Register (NTR4063).

### Study design

**Fig. 1:**
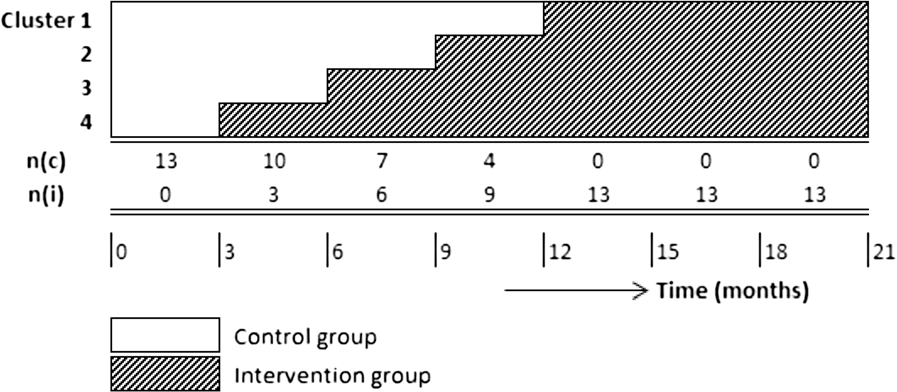
Schematic overview of the study process

The effects of the implementation of a PHR in maternity care on patients and professionals will be identified in a stepped-wedge, cluster-randomised, controlled trial. This type of trial is suitable for studying the effects of a new intervention, which is implemented at the cluster level, but experienced and measured by its impact on individuals [11, 12]. In essence, a stepped-wedge trial randomly allocates clusters to groups that crossover from a control condition to an intervention at different crossover points. In the present study, all healthcare professionals, working at 13 sites (11 community-based midwife practices and two hospitals, comparable in the number of patients) agreed to participate. Following baseline measurements, sites will be randomly assigned to four groups, containing three or four sites. The two hospitals are randomly allocated to group 1 and group 2; groups 3 and 4 contained only community-based midwife practices. The length of time between the two successive crossover points is 3 months. The total trial duration is 21 months. Figure 1 illustrates the study over time, including a pre-rollout period, four crossover points, and a post-rollout period. At the start of the study, all sites belong to the control group; at the end of the study, all sites belong to the intervention group. Data collection will be performed in all steps along the trial, coinciding with all four crossover moments.

### Outcome measures

The primary outcome measure in this study is health outcomes in maternity care. We will use a composite maternal/neonatal outcome measure: the total proportion of uncomplicated pregnancies/births. This includes the following conditions: gestational age at time of delivery is 37–41 weeks, spontaneous start of contractions, spontaneous rupture of membranes, no help during delivery, weight of neonate 5–95 %, APGAR score after 5 min > 7, and no serious postpartum haemorrhage.

Secondary outcome measures are the quality of care from the patients’ perspectives, collaboration among healthcare professionals, and the adoption of MyPregn@ncy by patients and professionals.

### Intervention

The intervention in the present study is the implementation of a PHR, named MyPregn@ncy, for each pregnant woman and her healthcare professionals. MyPregn@ncy is provided by www.mijnzorgnet.nl. The initial PHRs facilitated by MijnZorgnet have recently been used for women experiencing infertility and persons with Parkinson’s disease [13]. The use of a PHR in a maternity care setting is new in the Netherlands.

To ensure safe access to MyPregn@ncy, pregnant women register and log in using their personal DigiD, an identification code provided by the Dutch government. Healthcare professionals register and log in with their personal professional national electronic identification or with a username and password.

Once registered on the website, the pregnant woman owns the PHR. She decides who is granted access to her PHR and, therefore, is a member of her personal care team. She can invite all people (e.g. healthcare professionals and partner/relatives) she considers to be important for her health and for the care process during her pregnancy and birth.

Functionalities of the PHR MyPregn@ncy include the following: (1) communication with one or more care team members, (2) a diary (blogging feature), (3) a library (storage of important documents), and (4) interactive (medical) modules specifically developed for pregnant women. All team members in one PHR can access all fields in the PHR and can add, act, or react. All activities are logged, so the owner has full insight into all delivered input.

In addition to the PHR, the informational website www.mijnzwangerschap.org will be launched to support the introduction and use of MyPregn@ncy and the accompanying study.

To test the developed prototype of the PHR and design of the implementation strategy, we organised eight focus groups. Participants (n = 41) were recommended patient representatives from 16 hospitals and healthcare organisations across the Netherlands. A focus group interview guide was developed in conjunction with other researchers to ensure objectivity. Participants discussed when they would join a PHR, what was unclear about the PHR, what frightened them, and when a PHR had additional value to standard care. Focus groups were audiotaped and transcribed verbatim. Transcripts were read several times and analysed. The model of Cabana et al. [14] was used to analyse all focus group discussions in a systematic way. We categorised and identified six major themes from the focus group data: patient empowerment, safety and access, communication, culture change, legal aspects, and organisation. The results indicated that the information on the PHR must be secure and easy to use and that additional value lies in patient empowerment and online communication. Participants expressed concerns about accessibility for all patients (language and writing problems) and legal aspects (liability and privacy law). Overall, the message resulting from the focus groups showed that a PHR for pregnant women has value but must be a tool next to regular care instead of a replacement of care. Thanks to the input of the focus groups, we learned about barriers to and facilitators of a PHR and designed our communication about the study and the implementation strategy.

### Implementation strategy

MyPregn@ncy will be offered to the pregnant woman by the midwife practices or obstetrics clinics, depending on where she receives her care. To successfully offer the PHR, all healthcare professionals must indeed inform the pregnant woman about MyPregn@ncy, they must understand the use and possibilities of MyPregn@ncy (so that they can explain it correctly), and that they must have their own profile on www.mijnzorgnet.nl (so they can actively participate in MyPregn@ncy with the pregnant woman). To achieve optimal preparation and propagation of the tool, we will create various information leaflets and an informational and supporting website, www.mijnzwangerschap.org. Furthermore, we will visit each participating midwife practice and obstetric clinic to explain MyPregn@ncy and the study design.

In addition to the face-to-face information from their healthcare professionals, pregnant women will be informed of the PHR by local newspapers and by a short movie on digital information screens in the practices and clinics.

During all communication, it will be stated explicitly that the present implementation will be studied and evaluated. It will be emphasised that, at all times, researchers will not have the ability to directly access the PHR. Informed consent will be obtained from each participant. During the study, researchers will be available for questions and clarification for both pregnant women and healthcare professionals. Finally, involved healthcare professionals will regularly receive newsletters on the progress of the study.

### Measurements

Table 1 presents an overview of all measurements used.

**Table 1:** Overview of outcome measures with accompanying methods and measurements

Outcome measure	Study population	Method	Instrument	Time of measurement
Healthcare	All pregnant women in care in the area	Database	Perinatal Data Register	Five times, along all successive steps of the study
Quality of care	All pregnant women in care in the area	Survey	Questionnaire ReproQ	Five times, along all successive steps of the study
Collaboration	All maternity care healthcare professionals in the area	Survey	Social Network Analysis	Baseline and one post measurement
Adoption	All users of MyPregn@ncy	Log format, Survey, Interview	Log data, Questionnaire, Topic list	During and after implementation

The total proportion of uncomplicated pregnancies/births will be based on data from the Dutch Perinatal Registry (PRN). In the Netherlands, more than 95 % of all births and accompanying perinatal outcomes are centrally registered in this large national database [10].

The quality of care from the patients’ perspectives will be measured by an adapted version of the questionnaire ReproQ [15]. This new questionnaire has been developed to evaluate the whole maternity care, regardless of where the care is given [15, 16]. Development of this self-report questionnaire has been based on the 8-domain WHO Responsiveness model, including the following domains: dignity, autonomy, confidentiality, communication, prompt attention, social consideration, basic amenities, and choice and continuity [17]. The original questionnaire consists of two parts with largely identical questions: an antepartum questionnaire and a postpartum questionnaire. Due to considerations of time and response, we will use a combined version of both questionnaires in the present study, 6 to 10 weeks postpartum. Subgroups of participants in the study groups will be invited to complete the ReproQ in every phase of the stepped-wedge design. The results of the questionnaires will be calculated in domain scores and overall scores, ranging from one to four [15]. PRN data and ReproQ data will be collected during each step of the stepped-wedge study.

Collaboration between healthcare professionals in maternity care will be measured using Social Network Analysis (SNA). SNA is a quantitative methodology that measures and analyses connections between healthcare professionals in patient care [18]. It combines the concept of the sociogram (a visual representation of relationships in a social group) with elements of graph theory to analyse patterns of interaction among people in networks [18]. We will measure SNA two times in this study: a baseline measurement before the start of the intervention and one post measurement at the end of the study. Therefore, all healthcare professionals in maternity care in the area will be digitally invited for SNA. They will be asked to indicate with whom of their colleagues they had medically oriented contact concerning at least one patient in the last 6 months. The results will be illustrated in a sociogram, and various key network measures will be calculated, such as the number of contacts and centrality of each profession [19]. Finally, the adoption of MyPregn@ncy will be determined by a process evaluation during and after the implementation, according to Hulscher et al. [20]. This process evaluation will provide insight into the usage and appreciation of the various elements of the PHR by the pregnant women and their healthcare professionals. Barriers and facilitators to the use of MyPregn@ncy will be determined, using the theoretical framework of Cabana et al. [14]. Information will be gathered through semi-structured telephone and face-to-face interviews, and questionnaires. Log data will be used to measure the number of accounts and the number of logins per person. Qualitatively, we will conduct interviews based on a topic list with patients and healthcare professionals on their experiences with the intervention, including their perceptions of its utility, convenience, barriers, and facilitators to implementation, and options on long-term sustainability.

### Statistics

Sample size calculations have been based on an expected improvement of 47 % to 60 % in the proportion of uncomplicated births. Combined with an alpha of 0.05, 80 % power, and a design effect (correction factor due to a stepped-wedge design [21]) of 0.62, a total of 305 pregnant women is necessary for each step. With 13 sites and an expected uptake rate of 25 %, 84 pregnant women/step/site are needed to measure the effects.

Data analysis for the stepped-wedge design will be performed according to an intention-to-treat protocol at the end of the study period, once all sites have at least completed one 3-month intervention period. Individual PRN and ReproQ data will be combined with the different sites. To estimate the intervention effect on the outcome measures, a standard mixed-model approach will be used. The mixed model will incorporate fixed terms for intervention (before versus after), time (3-month period), as well as a correlation of repeated measurements over time as random effects. Hence, the analyses will have the element of a time-series analysis with multiple time points before and after the intervention. Analyses will be performed using SPSS (version 20.0 for Windows: SPSS Inc., Chicago, IL, USA).

Description of the network data by Social Network Analysis (SNA) involves visual analysis of network diagrams produced using a specific software program, NetDraw [22]. This software converts matrices of network data into diagrams and individual nodes using complex algorithms. We will use UNICET v6 [19] to construct the network and obtain network parameters and SPSS 20 (SPSS Inc., Chicago, IL, USA) for statistical analysis.

In addition to log data analysis, the interviews will be audiotaped and transcribed verbatim. The interviews will be independently coded by two persons based on the topic list.

## Discussion

The presented paper describes the design of a study in which a PHR will be offered to pregnant women and their healthcare professionals in order to improve health outcomes, to improve patients’ experiences with maternal care, and to achieve more collaboration between healthcare professionals. As far as we know, this study is the first to implement a PHR in maternity care. The study is expected to yield new information about the strengths, possibilities, and challenges to the implementation and usage of a PHR in maternal care settings. Results may lead to new insights and improvements in the quality of maternal and perinatal care.

The present study has several strong aspects. Earlier experiences with PHRs on the platform www.mijnzorgnet.nl have shown promise for more extensive use [13]. In addition, patient representatives have been consulted in the development of the implementation strategy. Furthermore, the choice of a stepped-wedge study design rather than a standard, randomised, controlled trial takes into account the actual different levels of implementation (cluster level) and experience and measurements (individual level).

We realise the study has some aspects that could have been more optimal. Ideally, the patient representatives should have included pregnant women. Furthermore, a pilot study preliminary to the present study would have contributed to a more detailed preparation. Finally, the study design has several limitations. We realise that a 3-month period for each step might be short for the measurement of an effect. However, we made this choice deliberately, balancing among the number of steps, length of each interval, and total length of the study. Our overall focus in this choice was to perform a well-designed study in a practical time setting. Additionally, the study region has only two hospitals. Due to the multidisciplinary aspect of the intervention, hospitals should be present early in the intervention group. Therefore, we have chosen to randomly allocate the two hospitals to group 1 and group 2, whereas groups 3 and 4 contained only community-based midwife practices. By doing this, we are well aware of the potential underlying differences in treatment effect among the patient populations. Therefore, we will focus special attention for this confounding effect in the analyses.

This study is important because of the recently expressed strong wish for an easy and accessible PHR for each citizen in 2020 by the leading healthcare players in the Netherlands. The results of the study can help facilitate this wish and perfectly connects to the current developments in Dutch healthcare.

### Trial status

Community-based midwife practices and hospitals have been included. The study is currently recruiting participants, i.e. individual clients and professionals. The estimated study completion date is April 2016.

## Abbreviations

PHR: personal health record

## References

[1] Tang PC, Ash JS, Bates DW, Overhage JM, Sands DZ (2006). Personal health records: definitions, benefits, and strategies for overcoming barriers to adoption. J Am Med Inform Assoc.

[2] Tenforde M, Jain A, Hickner J (2011). The value of personal health records for chronic disease management: what do we know?. Fam Med.

[3] Osborn CY, Mayberry LS, Mulvaney SA, Hess R (2010). Patient web portals to improve diabetes outcomes: a systematic review. Curr Diab Rep.

[4] Silvester BV, Carr SJ (2009). A shared electronic health record: lessons from the coalface. Med J Aust.

[5] Zwangerschap en geboorte S (2009). A good start: safe care during pregnancy and birth (Een goed begin, Veilige zorg rond zwangerschap en geboorte).

[6] EURO-PERISTAT (2013). European perinatal health report. The health and care of pregnant women and babies in Europe in 2010.

[7] Bosch M, Faber MJ, Cruijsberg J, Voerman GE, Leatherman S, Grol RP (2009). Review article: effectiveness of patient care teams and the role of clinical expertise and coordination: a literature review. Med Care Res Rev.

[8] Amelink-Verburg MP, Buitendijk SE (2010). Pregnancy and labour in the Dutch maternity care system: what is normal? The role division between midwives and obstetricians. J Midwifery Womens Health.

[9] Offerhaus PM, Hukkelhoven CW, de Jonge A, van der Pal-de Bruin KM, Scheepers PL, Lagro-Janssen AL (2013). Persisting rise in referrals during labor in primary midwife-led care in the Netherlands. Birth.

[10] Stichting Perinatale Registratie Nederland U (2016). Perinatal care in The Netherlands 2014 (Perinatale zorg in Nederland 2014).

[11] Copas AJ, Lewis JJ, Thompson JA, Davey C, Baio G, Hargreaves JR (2015). Designing a stepped wedge trial: three main designs, carry-over effects and randomisation approaches. Trials.

[12] Hemming K, Haines TP, Chilton PJ, Girling AJ, Lilford RJ (2015). The stepped wedge cluster randomised trial: rationale, design, analysis, and reporting. BMJ..

[13] Aarts JWM, Vennik F, Nelen WLDM, van der Eijk M, Bloem BR, Faber MJ (2015). Personal health communities: a phenomenological study of a new health-care concept. Health Expect.

[14] Cabana MD, Rand CS, Powe NR, Wu AW, Wilson MH, Abboud PA (1999). Why don’t physicians follow clinical practice guidelines? A framework for improvement. JAMA.

[15] Scheerhagen M, van Stel HF, Birnie E, Franx A, Bonsel GJ (2015). Measuring client experiences in maternity care under change: development of a questionnaire based on the WHO responsiveness model. PLoS One.

[16] van der Kooy J, Valentine NB, Birnie E, Vujkovic M, de Graaf JP, Denktaş S (2014). Validity of a questionnaire measuring the world health organization concept of health system responsiveness with respect to perinatal services in the Dutch obstetric care system. BMC Health Serv Res..

[17] Gostin L, Hodge JG, Valentine N, Nygren-Krug H (2003). The domains of health responsiveness – a human rights analysis.

[18] Scott J, Tallia A, Crosson JC, Orzano AJ, Stroebel C, DiCicco-Bloom B (2005). Social network analysis as an analytic tool for interaction patterns in primary care practices. Ann Fam Med.

[19] Borgatti SP, Everett MG, Freeman LC (2002). UNIcet for Windows: software for social network analysis.

[20] Hulscher ME, Laurant MG, Grol RP (2003). Process evaluation on quality improvement interventions. BMJ Qual Saf.

[21] Hussey MA, Hughes JP (2007). Design and analysis of stepped wedge cluster randomized trials. Contemp Clin Trials.

[22] Borgatti S (2002). Netdraw: Graph Visualization Software.

